# Early Development of Clinical Reasoning Through Virtual Patient Simulation: Nursing Students’ Perceptions of Collaborative Decision-Making

**DOI:** 10.3390/nursrep16050152

**Published:** 2026-04-30

**Authors:** Leila Sales, Maria Ferreira, Raquel Pereira, Isabel Lucas, Rita Marques, Inês Bento

**Affiliations:** 1 Portuguese Red Cross Higher School of Health, 1300-125 Lisbon, Portugal; mferreira@esscvp.eu (M.F.); raquelpereira@esscvp.eu (R.P.); ilucas@esscvp.eu (I.L.); rmarques@esscvp.eu (R.M.); ibento@esscvp.eu (I.B.); 2 Health Sciences Research Unit: Nursing, Nursing School of Coimbra, 3045-043 Coimbra, Portugal; 3 Instituto de Saúde Ambiental (ISAMB), Faculty of Medicine, University of Lisbon, 1649-028 Lisbon, Portugal; 4 Centre for Interdisciplinary Research in Health, Portuguese Catholic University, 1649-023 Lisbon, Portugal; 5 Nursing Research Innovation and Development Centre of Lisbon (CIDNUR), School of Nursing, Universidade de Lisboa, 1990-096 Lisbon, Portugal

**Keywords:** simulation, computer simulation, virtual patients, clinical reasoning, critical thinking, clinical decision-making, nursing education, patient safety

## Abstract

Simulation is increasingly recognised as a strategic approach in nursing education for developing clinical competencies within safe learning environments. However, there is limited understanding of how virtual patient simulation supports the early development of clinical reasoning from the perspective of nursing students. **Aim**: To explore the perceptions of first-year undergraduate nursing students regarding the development of clinical reasoning and collaborative decision-making through virtual patient simulation. **Methods**: A qualitative, descriptive, and exploratory design was adopted. Semi-structured focus groups were conducted with 73 first-year undergraduate nursing students. Data were analysed using thematic content analysis following Bardin’s approach. **Results**: Students perceived virtual patient simulation as a meaningful and high-impact learning strategy. Realism, interactivity, and group collaboration emerged as key strengths. Engagement with dynamic clinical scenarios supported the integration of theoretical knowledge into practice, enhanced prioritisation skills, and promoted structured clinical reasoning. Collaborative learning facilitated shared reflection and collective problem-solving, while immediate feedback enabled learning through error within a psychologically safe environment. Participants also reported increased confidence and autonomy in decision-making. At the same time, students identified limitations related to software constraints and the alignment between automated assessment and their reasoning processes. **Conclusions**: Group-based virtual simulation appears to support the early structuring of clinical reasoning, extending beyond technical skill acquisition to foster reflective and collaborative practice. Its educational value, however, depends on intentional curricular integration and strong pedagogical alignment including structured facilitation, alignment between assessment and learning objectives, and opportunities for guided reflection. These findings contribute to a process-oriented understanding of how novice learners make sense of clinical reasoning in simulated contexts.

## 1. Introduction

Clinical reasoning is widely recognised as a core competence in healthcare education, given its direct implications for decision-making and patient safety. Failures in clinical reasoning are associated with a substantial proportion of diagnostic errors, estimated to account for approximately 10–15% of errors in healthcare delivery [1]. These findings highlight the importance of adopting educational strategies that intentionally support the development of clinical reasoning, particularly from the early stages of professional training.

Despite its recognised importance, the development of clinical reasoning in early-stage nursing education remains a significant pedagogical challenge. Evidence suggests that novice students often struggle to interpret clinical cues, prioritise information, and integrate theoretical knowledge into practice, tending instead to rely on fragmented or task-oriented approaches rather than structured reasoning processes [2,3,4]. This difficulty has been associated with limited clinical exposure, underdeveloped cognitive schemas, and insufficient opportunities to engage in authentic decision-making situations during the initial stages of training [3,5]. Importantly, these limitations have been identified as contributing factors to difficulties in transitioning from theoretical learning to clinical practice [2,4], representing a well-documented educational problem in nursing education that justifies the need for targeted pedagogical interventions.

Clinical reasoning is understood as a complex, contextual, and dynamic process that underpins decision-making in nursing. It can be understood through theoretical models that describe both its structure and the underlying cognitive processes. Among these, the Clinical Reasoning Cycle offers a systematic approach, describing reasoning as an iterative process that includes considering the patient’s situation, collecting and processing information, identifying problems, setting goals, implementing interventions, evaluating outcomes, and reflecting on the process [6,7]. Complementarily, Tanner’s Clinical Judgment Model [4] conceptualises clinical reasoning as an interpretive process involving four interrelated dimensions: noticing (perceiving relevant clinical cues), interpreting (making sense of the information), responding (taking clinical action), and reflecting (reflecting on the action). This model highlights the role of experience, prior knowledge, and context in the development of clinical judgement.

The integration of these models allows clinical reasoning to be understood as both a structured and interpretive process, through which students progressively develop the ability to recognise patterns, establish relationships between data, and make informed decisions. Additionally, these perspectives can be complemented by Social Cognitive Learning Theory [8], which emphasises the role of observational learning, social interaction, and self-efficacy in shaping cognitive processes. Within simulation environments, learners actively construct knowledge through interaction with scenarios, feedback, and peers, reinforcing both cognitive and metacognitive dimensions of reasoning.

In this context, virtual simulation represents a particularly relevant pedagogical strategy, as it creates safe and interactive environments that enable students to iteratively engage with the different stages of clinical reasoning. By exposing students to dynamic clinical scenarios, simulation promotes the mobilisation of noticing and interpreting processes, as well as decision-making (responding) and reflection (reflecting), aligning with the assumptions of the theoretical models mentioned above. Furthermore, simulation environments grounded in social cognitive principles enable learners to observe modelled behaviours, engage in collaborative reasoning, and develop self-regulation strategies, which are essential for the consolidation of clinical reasoning skills. Thus, the use of virtual simulation in the early stages of education may support engagement with the early structuring of clinical reasoning, fostering the early development of cognitive and metacognitive skills essential for professional practice.

Nursing education faces ongoing challenges in preparing students for complex clinical situations, particularly in developing clinical reasoning and safe decision-making, areas in which simulation-based approaches have been increasingly explored [9,10]. Within this context, clinical simulation has become an essential pedagogical approach, offering controlled environments where students can practice, reflect, and learn without compromising the well-being of real patients, an increasingly recognised ethical imperative [11,12,13]. Opportunities to make mistakes in psychologically safe environments promote reflection, immediate feedback, and the consolidation of knowledge and skills [14].

Evidence suggests that simulation-based learning promotes active knowledge construction, strengthens clinical reasoning by exposing learners to a wide range of scenarios, and improves communication and teamwork [15]. These competencies are essential for sound clinical decision-making [16,17]. In addition, collaborative simulation approaches can further support shared problem-solving and encourage more structured reasoning processes [18].

Advances in digital technology have expanded the possibilities of simulation, with virtual patient platforms (VPs) emerging as innovative tools capable of replicating complex clinical interactions. VPs represent a form of simulation capable of replicating complex clinical interactions based on scenarios that reflect real-life situations, enabling students to develop the knowledge and skills required to manage patient care [19,20]. Body Interact^®^ is one such platform, enabling students to assess virtual patients in real time, interpret dynamic clinical data, implement interventions, and observe physiological responses. By placing students at the centre of the learning process, virtual simulation encourages the mobilisation of cognitive, decision-making, communication, and psychomotor skills across varied clinical contexts [21]. In addition, Body Interact^®^ (Coimbra, Portugal) provides a safe and flexible learning environment where students can repeatedly practice clinical scenarios without risk to real patients, enhancing confidence and reducing anxiety. Its adaptive nature allows learners to experience the consequences of their decisions, promoting reflective practice and deeper clinical reasoning.

Within this context, virtual simulation may support different dimensions of clinical reasoning, such as cue recognition, data interpretation, prioritisation of interventions, and reflective evaluation of decisions, which informed both the design of the learning activity and the analytical lens adopted in this study [22].

Research has increasingly documented the educational benefits of virtual patient simulation, including improvements in clinical reasoning, critical reflection, collaborative decision-making, and curriculum integration [23,24,25]. Experimental evidence also suggests that students exposed to virtual simulation demonstrate greater knowledge gains and higher satisfaction compared to those engaged in lower fidelity approaches [21]. However, much of the existing literature has focused on learning outcomes, performance indicators, or student satisfaction [26,27] with comparatively less attention given to how students experience and construct clinical reasoning processes during the early stages of their education. This represents an important gap, as early learning experiences may play a critical role in shaping how reasoning is initially structured, before more complex clinical exposure occurs.

Furthermore, there is limited understanding of how novice students interpret simulation experiences and how these experiences contribute to the initial organisation of reasoning processes, particularly in collaborative learning contexts. Addressing this gap is essential to guide pedagogical design and to ensure that simulation is both effective and suitable for learners at early stages of development.

Despite growing evidence supporting the pedagogical value of virtual simulation, less is known about how early exposure to these technologies may influence the development of clinical reasoning, particularly during the initial stages of nursing education. Therefore, this study not only explores students’ perceptions but also seeks to contribute theoretically by clarifying how reasoning processes begin to be structured in early training, and practically by guiding the design of simulation-based learning activities tailored to novice learners.

This study contributes new knowledge by focusing specifically on first-year students, a group that has been underexplored in the literature, and by examining how early encounters with virtual simulation shape the initial structuring of reasoning processes and engagement with clinical decision-making [17].

The focus on first-year exposure is theoretically relevant because it captures a formative stage in which students are beginning to construct cognitive frameworks for clinical reasoning, before habits and patterns become consolidated. Early pedagogical experiences may therefore play a critical role in shaping how students interpret clinical cues, approach problem-solving, and engage in collaborative reasoning [28].

Understanding students’ perceptions may therefore provide important insights into the formative role of virtual simulation and inform more intentional curricular design.

The research questions guiding this study were: (1) How do first-year nursing students perceive the role of virtual patient simulation in the development of clinical reasoning? and (2) How do these students perceive the contribution of collaborative simulation to their decision-making processes?

Accordingly, this study aimed to explore the perceptions of first-year undergraduate students regarding the development of clinical reasoning and collaborative decision-making through virtual patient simulation.

In doing so, it provides new insights into how clinical reasoning begins to develop in novice learners within collaborative virtual simulation, informing early nursing education design.

## 2. Materials and Methods

### 2.1. Study Design

An exploratory, qualitative, descriptive design was adopted. The aim of this study is to explore the perceptions of first-year undergraduate nursing students regarding the development of clinical reasoning and collaborative decision-making through virtual patient simulation.

All students had the opportunity to work in groups to complete eight Body Interact cases, each lasting 20 min (predefined by the software), related to fundamental nursing care and medical-surgical nursing. Following the completion of each case, a 40 min debriefing session was conducted.

Given the educational context of the study, particular attention was paid to reflexivity, especially regarding the potential influence of researchers involved in the pedagogical process. The research team critically reflected on their roles, relationships with participants, and possible sources of bias throughout the study design, data collection, and analysis phases.

### 2.2. Participants

The study involved 73 first-year undergraduate nursing students from a Higher School of Health Sciences in Lisbon who had successfully completed a course unit. Given the pedagogical organisation of the course and the intention to capture a broad range of shared and divergent experiences, all eligible students were invited to participate. Rather than aiming for statistical representativeness, the sample was purposive and information-oriented, in line with qualitative research principles. We acknowledge that participants belonged to a single cohort, institution, and educational intervention. As such, the findings are contextually situated and should be interpreted within this specific setting. The aim of the study is therefore not generalisation, but to generate theoretically and pedagogically relevant insights that may be transferable to similar contexts.

The final sample size was determined by data saturation, which was reached when additional data confirmed previously collected findings and no longer yielded new insights.

### 2.3. Eligibility Criteria

Inclusion criterion:

Attendance of the *Pathology and Clinical Reasoning* course under continuous assessment.

Exclusion criterion:

- Undergraduate nursing students with previous experience using virtual patient software.

### 2.4. Data Collection

The study was conducted between October 2024 and May 2025, aligning with the academic term of the *Pathology and Clinical Reasoning* course unit in the first year of the nursing degree.

Data collection was conducted through a focus group, an appropriate technique for understanding socially constructed perceptions and exploring interaction among participants [29,30,31]. This methodology enables the identification of shared reasoning, differences in opinion, and processes of co-constructing meaning—elements that are particularly relevant to the study of clinical reasoning and group decision-making [13].

Six focus groups were conducted, each comprising 8 to 13 students, held in person at the facilities of the School of Health Sciences. Each session lasted approximately 50 min.

Although the group size exceeded typical qualitative recommendations, it was intentionally aligned with the collaborative nature of the learning activity, allowing the capture of interactional dynamics and shared meaning-making [29]. To address potential limitations, the moderator promoted balanced participation, encouraged quieter students, and used probing questions, while an observer monitored group dynamics. Despite the larger group size, depth and richness were ensured through active moderation strategies, including targeted probing, encouragement of diverse participation, and attention to interactional dynamics, allowing both individual perspectives and collective meaning-making to emerge.

The moderator was a researcher with experience in qualitative methods, while a second researcher acted as an observer, ensuring time management, audio recording, and the documentation of relevant aspects of group dynamics. The moderators were not responsible for the formal assessment of the participating students. Participants were explicitly informed that their decision to participate or the nature of their responses would have no impact on their academic evaluation. Focus groups were conducted after completion of the course unit assessment, further reducing perceived pressure or social desirability bias.

A semi-structured guide was used to moderate the focus groups. It was developed by the research team and aligned with the COREQ [30] criteria for rigour and transparency. The guide was organised into sequential thematic blocks and included open-ended questions focusing on:

1. *How students perceive the experience of developing clinical reasoning and group decision-making during simulations with virtual patients;*
2. *The contribution of group-based, simulation case-solving to the development of clinical reasoning and decision-making;*
3. *The opportunities identified by participants when solving cases with virtual patients in a group setting;*
4. *The perceived disadvantages of solving cases using virtual patients in a group setting;*
5. *The importance attributed to instructors’ involvement in facilitating scenario-based case resolution using the software, promoting clinical reasoning, and supporting collaborative decision-making.*

The guide was applied in a flexible and interactive manner, allowing student interaction to shape the depth of discussion and enabling the introduction of follow-up/probing questions whenever appropriate. During the sessions, efforts were made to foster a safe, supportive, and non-evaluative environment, facilitating the participation of all group members and ensuring freedom of expression.

Additionally, the moderator adopted a reflexive stance, remaining attentive to verbal and non-verbal cues, encouraging diverse viewpoints, and avoiding leading questions. Reflexive field notes were recorded after each session to document contextual influences, researcher assumptions, and group dynamics that could shape data interpretation.

Before the start of each focus group, participants were informed about the objectives, procedures, participation rules, and confidentiality safeguards. Each session was audio-recorded with informed consent.

Each interview was assigned a unique identification number according to chronological order. A categorization system was established to organise exploratory data, aiming to structure and prepare the content for subsequent analysis.

The choice of focus groups as the data collection technique was grounded in the collaborative nature of the phenomenon under study, namely, the development of clinical reasoning and group decision-making. This methodological approach makes it possible to understand how students collectively articulate ideas, observe and respond to peers’ contributions, and build shared lines of reasoning, generating richer data than individual interviews would be likely to produce.

Moreover, the group dynamics reflected the learning environment fostered by the established pedagogical approach, further reinforcing the suitability of this technique for the object of study.

### 2.5. Data Saturation

Saturation was considered achieved when successive focus groups no longer produced meaningful new experiential insights, and discussions began to consistently echo shared experiences within the same educational context. In this study, saturation is thus conceptualised as the convergence and recurrence of experiential narratives, rather than the exhaustive identification of distinct conceptual categories.

The decision to conclude data collection was based on ongoing comparative analysis across focus groups, which demonstrated consistency in participants’ meanings and sufficient depth to address the exploratory aims of the study [29].

### 2.6. Methodological Rigour

The qualitative study was conducted in accordance with the rigour criteria proposed by Guba and Lincoln [32], ensuring the quality of the findings across the following dimensions:

Credibility

It was ensured through the following measures:

- Researcher triangulation during data analysis;
- Cross-validation of emerging categories through comparison across different focus groups;
- Regular analytical discussions among the researchers to clarify interpretations and minimise bias;
- Ongoing reflexive engagement by the research team, critically examining how their backgrounds, assumptions, and roles within the educational context could influence data interpretation.

Transferability

A detailed description of the teaching context, participant characteristics, and the process of using the Body Interact^®^ software allows other researchers to assess the applicability of the findings to similar contexts.

Rather than claiming broad generalisability, this study provides a “thick description” of the educational context and learning processes, enabling readers to determine the relevance and transferability of the findings to comparable educational settings.

Dependability

Systematic records of the analytic process were maintained, including field notes, categorization decisions, and successive revisions of interpretations. These records ensure the auditability of the process.

Confirmability

To reduce the influence of researcher bias, the following were used:

- Reflexive notes throughout data collection and analysis;
- Independent review of the categories by different members of the research team;
- Integrative discussion to achieve interpretive consensus;
- Explicit consideration of the researchers’ positionality and its potential impact on analytic decisions, documented within the analytic diary.

Focus Group-Specific Ethical Considerations

Collective confidentiality rules were reinforced, emphasising to participants that, although anonymity would be ensured during analysis, the researcher could not control what other group members might share outside the session. All students provided written informed consent, were informed that participation was voluntary, and were free to withdraw at any time without any impact on their academic progress.

### 2.7. Data Treatment and Analysis

The data were analysed using a qualitative approach, specifically thematic content analysis following Bardin’s method [33]. The process involved initial open coding and subsequent thematic categorization.

Following the focus group sessions, the audio recordings were fully transcribed by the researchers, and the resulting data were subjected to thematic content analysis according to Bardin’s method, comprising three main phases: pre-analysis, material exploration, and treatment/inference/interpretation.

Coding was performed manually by the coders. To strengthen scientific rigour, two researchers independently coded the data and subsequently compared their codes and interpretations. In cases of disagreement, the team met for reflective discussion until interpretive consensus was reached. The categorization process was therefore iterative and collaborative, ensuring conceptual coherence between the findings and the study’s objectives.

Throughout the analytic process, reflexive discussions were held to critically examine how researchers’ perspectives and prior involvement in the educational context might shape coding decisions and theme development.

Given the interactive nature of focus groups, the analysis included not only individual meaning units (significant statements) but also patterns of group interaction, namely: agreement, disagreement, complementary reasoning, and moments of co-construction of meaning. In parallel, field notes and reflexive notes were produced throughout data collection and analysis, enabling the tracking of methodological and analytical decisions and strengthening dependability and confirmability.

To ensure auditability, a structured record (analytic diary) was maintained, including: code definitions, examples drawn from the focus groups, decisions to merge or split themes, and the evolution of emerging categories. The final thematic interpretation was validated by the research team through cross-checking narratives and conceptual consolidation until the seven categories reported in the results were stabilised.

### 2.8. Ethical Considerations

This study adhered to the ethical principles outlined in the Declaration of Helsinki and received approval from the Higher School of Health Ethics Committee (Approval No. 12/2024).

All data were securely stored in digital format, anonymized and protected by password-restricted access, available only to the research team. Informed written consent was obtained from all participants, who received a detailed information sheet explaining the study’s objectives, voluntary nature, and their right to withdraw without any academic consequences. Given that the study was conducted within a course context, it was explicitly clarified that participation or non-participation would have no influence on students’ grades, assessment, or relationship with faculty members. Consent was obtained by a member of the research team not directly involved in students’ evaluation, ensuring that participation was free from perceived coercion or undue influence.

Refusals to participate were respected confidentially and did not affect students’ academic standing or evaluation. Students were also informed that they could withdraw at any stage of the study without providing justification and without any academic penalty.

## 3. Results

Seventy-three first-year students enrolled in a Bachelor’s degree in Nursing at a School of Health Sciences in the Lisbon region took part in this study. Participants ranged in age from 19 to 40 years; 90.4% were female and 9.6% were male.

Data processing enabled the deconstruction of the discourse, followed by its organisation, systematisation, and analysis, with the aim of selecting, grouping, simplifying, and transforming the data. Beyond this descriptive organisation, the analytic process sought to interpret how students construct meaning around their learning experiences, particularly in relation to clinical reasoning and collaborative decision-making. This process yielded categories and subcategories reflecting students’ perceptions of the development of clinical reasoning and group decision-making when using simulation with virtual patients.

From the qualitative analysis of the data, based on the narratives of nursing students (coded with the letter “G” followed by the interview number), seven categories were identified, two of which were further divided into specific subcategories:

- Approximation to clinical reality;
- Dynamism, interactivity, and teamwork;
- Translating theory into practice;
- Student empowerment and self-awareness;
- Learning through error;
- Software limitations;
- Pedagogical limitations.

- Approximation to clinical reality

Students reported a strong sense of realism when using the software, particularly during the clinical data collection process, with realism emerging not merely as a feature of the simulation but as a cognitive and emotional anchor that promotes deeper engagement in clinical reasoning. In this sense, realism appears to support early-stage reasoning processes by enabling cue recognition and situational interpretation, aligning with the “noticing” and “interpreting” dimensions of clinical judgement, while also facilitating the contextualisation of clinical information and decision-making within meaningful, practice-oriented scenarios. The relevance of interacting with the virtual patient and their family was highlighted, as was the opportunity to experience situations that are typically absent in classroom settings, as illustrated by students from different groups: “*Home visits, during data collection (…) we never had the opportunity to practice in class.*” (G1); “*We think that, since the virtual mannequin is always more realistic*” (G2); “*(…) we can see how [the person] really is, how they are reacting, or whether they are sweating, for example.*” (G3); This perceived realism appears to enable students to interpret clinical cues, anticipate patient responses, and situate their decisions within a meaningful and practice-oriented context, as reflected in statements such as: “*You can really see it (…) I think it affects us (…)*” (G4); “*Unpredictable situations that occur during the case are closer to reality.*” (G5); “*(…) he [the virtual patient] would always end up showing the effects—for example, if we administered the wrong medication, the patient could become unconscious.*” (G6); “*Visually, we can associate what we are seeing with the pathology or condition being presented—I think that is very good.*” (G2).

II. Dynamism, Interactivity, and Teamwork

The simulation was described as a dynamic and stimulating teaching strategy that promotes interaction among participants and teamwork. This suggests that dynamism functioned not only as an engagement factor, but as a mechanism supporting sustained cognitive involvement in reasoning processes. Students stated, *“It is a dynamic way of learning that is not boring*” (G1), and also highlighted the complementarity within the groups. Importantly, teamwork emerged not only as a structural feature of the activity but as a mechanism for the co-construction of clinical reasoning. “*Colleagues complemented each other (...) some would say things that were then complemented by other groups*” (G2). “*Working in a group helps us think of things we had not considered and complements our reasoning.*” (G1). These findings indicate that clinical reasoning is socially mediated and distributed across group interactions, reflecting learning processes consistent with social cognitive perspectives. Through dialogue, negotiation, and exposure to multiple perspectives, students collectively refined their interpretations of clinical data, suggesting that reasoning was distributed across the group rather than individually contained. “*Obviously, if it is done in a group, it is much richer (…) it is good to hear everyone’s opinion.*” (G5).

Alongside the value attributed to peer cooperation, students also emphasised the importance of the teacher’s presence during case resolution through virtual patient simulation. The instructor was interpreted not merely as a facilitator, but as a mediator of reflective thinking and group reasoning processes, as highlighted: *“Teachers encourage us to think together*” (G2; G3); “*The teacher can also point out certain situations, give examples,* etc.” (G1).

The teaching–learning approach based on solving clinical cases with virtual patients supported teamwork, which students recognised as fundamental for sharing experiences and knowledge. “*Teamwork, because we can work cooperatively to solve the cases*” (G4; G6); “*We were able to plan everything together and then execute it in the simulator*” (G3).

III. Translating Theory into Practice

This category, which emerged from the data, was subdivided into two subcategories:

(a) Integrating Theory into Practice

Participants highlighted the integration of content learned in theoretical courses with its application in a simulated practical context. This integration was not described as a simple transfer of knowledge, but as a process of recontextualization, in which previously acquired theoretical concepts are actively mobilised, adapted, and interconnected within complex clinical scenarios. “*We also developed the care plans (...) we articulated and integrated them with what we had already covered in other subjects, such as anatomy or fundamentals*” (G2); “*In theory we know the pathologies (…) the program [virtual patient software] helps a lot in recognizing what was taught in the classroom*” (G3); “*In the program [virtual patient software] the person appears with more than one pathology, and that helps to connect the material.*” (G6).

(b) Prioritising Care in the Context of Patient Complexity

The simulator was perceived by the student groups as an opportunity to experience more complex clinical situations. This reflects an emerging capacity for interpretive judgement, where students’ narratives indicate that exposure to multiple and interacting pathologies supported the development of prioritisation skills, requiring them to weigh competing clinical demands and make context-sensitive decisions. “*In the program, the person appears with more than one pathology (...) and it helps us understand what it is a priority to do.*” (G6); “*It helped me truly understand the direction we need to take, how we should proceed, how we should act with the patient, and which key questions we need to ask. I think that was a real added value.*” (G3).

IV. Student Empowerment and Self-Awareness

The findings showed that group simulation contributed to strengthening students’ confidence and autonomy in the clinical decision-making process. This sense of empowerment appears to be closely linked to increased self-awareness regarding their own reasoning processes and clinical judgement. “*I gained more confidence*” (G3); “*We can now collect data better and understand what is important and a priority (…) I feel better prepared*” (G4). Rather than reflecting only increased confidence, these accounts suggest the emergence of a more reflective stance, in which students begin to recognise their role as active decision-makers. “*An opportunity to develop my own reasoning*” (G1). Increased confidence appears to signal the emergence of metacognitive awareness, as students begin to monitor and evaluate their own reasoning processes.

V. Learning Through Error

Data analysis indicated that students perceived the simulation as promoting meaningful learning, supported by the opportunity to learn from mistakes and by access to immediate feedback. Importantly, errors were not framed as failures, but as integral components of an iterative learning process. “*We failed quite a lot, but with clinical reasoning we can now collect data and understand what is important*” (G6). Immediate feedback appears to function as a trigger for the re-evaluation of clinical decisions, enabling students to recalibrate their reasoning and deepen their understanding of clinical priorities. “*We immediately get feedback on what we failed to do—what we failed to assess and what we failed to carry out. (…) it allows us to learn from mistakes.*” (G3); “*(…) sharing knowledge and learning from mistakes.*” (G4).

VI. Software Limitations

Despite the acknowledged benefits, the findings also highlighted two categories of limitations related to the software. These limitations were not only described in functional terms but were interpreted by students as constraints that may influence the alignment between simulated actions and professional nursing practice.

(a) Limited Suitability for Nursing Practice

At certain points, students identified gaps in the software, specifically the inability to operationalize certain nursing interventions: “In the software/program, important elements of nursing interventions that we perform are missing.” (G3); “Not being able to carry out certain actions [in the software] confuses us.” (G5). Students’ responses suggest a perceived gap between the scope of the simulator and the complexity of nursing interventions, which may affect the authenticity of the learning experience.

(b) Final Score Misaligned with Performance

In students’ perceptions, the final performance score for scenario resolution was at times misaligned with their decision-making and performance. Students prioritised and implemented certain interventions in the virtual environment that were not reflected as priorities or deemed necessary in the final score presented by the software. “*We do not consider that the software itself assesses our decision-making well.*” (G4); “*The program [software] lowered the grade by almost 50% after implementing an intervention that the software considered non-priority.*”

This misalignment was interpreted as a source of tension between students’ clinical reasoning and the system’s evaluative logic, potentially undermining the perceived validity of feedback. This topic highlights the difficulty of capturing interpretive judgement through standardised scoring systems.

VII. Pedagogical Limitations

With regard to pedagogical strategies, students identified several limitations. These constraints were interpreted as factors that may shape the depth of engagement and individual participation within the learning process. “*If we had practiced for more time with the teacher, it would have been better*” (G1); “*Each student should be able to contribute in each case*” (G1); “*Our cohort is very large and could have been divided into more groups, so that there would be fewer students per group.*” (G2). Students’ narratives suggest a need to balance collaborative learning with opportunities for individual involvement, highlighting the importance of pedagogical structuring in maximising the benefits of simulation.

Overall, the categories should not be understood as isolated pedagogical benefits, but as interconnected dimensions of a broader learning process in which simulation mediates the development of clinical reasoning through interaction, reflection, and contextualised decision-making.

## 4. Discussion

The findings of this study indicate that nursing students perceive virtual patient simulation as a meaningful pedagogical tool for decision-making, both individually and collaboratively. Importantly, these findings reflect students’ perceptions rather than objective measures of competence. The predominance of positive perceptions should therefore be interpreted with caution, particularly given the educational context in which data were collected, where relational and contextual factors may have influenced responses. These results are consistent with the literature highlighting the complexity and centrality of clinical reasoning in healthcare practice [34].

This study contributes to the literature by providing a process-oriented understanding of how first-year students engage with virtual simulation, an area less explored in research that has predominantly focused on advanced learners or outcome-based measures. Rather than demonstrating learning gains, the findings highlight how students construct meaning around simulation experiences and perceive engagement with reasoning-related processes. This focus on novice learners aligns with Benner’s model of skill acquisition, in which early-stage learners rely on context-dependent experiences to begin structuring clinical understanding, progressing from rule-based reasoning towards more integrated forms of practice [2].

The results align with established theoretical frameworks. Realism has been widely recognised as a key factor supporting immersion and engagement [12,13], while teamwork reflects the socially constructed nature of clinical reasoning [35,36]. Learning through simulation is consistent with experiential and reflective learning theories, emphasising action, feedback, and reflection [37,38]. However, the predominantly positive tone of participants’ accounts may also reflect contextual influences, reinforcing that findings should be interpreted as perceptions rather than demonstrable outcomes. In particular, the processes described by students resonate with Tanner’s Clinical Judgment Model, especially the early phases of noticing and interpreting, as learners begin to identify relevant cues and construct meaning from evolving clinical situations [4].

Simulation as a socio-cognitive learning environment

The findings support an understanding of virtual simulation as a socio-cognitive and reflective learning environment, rather than merely a technological tool. In particular, realism appears to function not only as a motivational feature but as a condition supporting cognitive immersion and engagement with clinical cues, evolving information, and situated decision-making. Plackett et al. [39] confirm that this type of simulation supports learning related to the collection and interpretation of clinical data, although such effects were not directly measured in the present study. From a theoretical perspective, this aligns with Tanner’s notion of noticing [4], as realism appears to scaffold students’ ability to attend to clinically relevant information within context.

Students also emphasised the importance of interacting not only with virtual patients but also with contextual elements such as family members and time constraints, reinforcing multiple dimensions of fidelity [40]. Emotional engagement and exposure to unpredictable scenarios further suggest that simulation may support preparation for managing uncertainty and pressure in clinical practice [41], as well as relational and emotional competencies [42]. In this sense, fidelity extends beyond representational accuracy, enabling interpretation, prioritisation, and reflection. Such experiences may contribute to the gradual development of clinical judgement, as described by Tanner, by encouraging learners to move beyond isolated cues towards more coherent interpretations of complex situations [4].

Collaboration and co-construction of reasoning

Dynamism, interactivity, and teamwork emerged as central to the learning experience. Beyond enhancing participation, teamwork enabled reasoning to be externalised, negotiated, and refined through dialogue. This aligns with Theobald et al. [43], who identify teamwork as both a facilitator and an outcome of simulation, and with Mohammadi-Shahboulaghi et al. [34], who emphasise the role of collaboration in managing complex clinical situations. These findings are also consistent with Bandura’s Social Cognitive Theory, which emphasises the role of social interaction, observation, and shared experience in shaping cognitive processes and learning [8].

The instructor’s role was also highlighted as essential in mediating learning, facilitating reflection, and supporting critical analysis, consistent with the INACSL Standards of Best Practice [12]. These findings reinforce that the educational value of simulation lies not only in the technology, but in the interaction between digital environments, peer collaboration, and pedagogical facilitation. Within this framework, instructors can be understood as key agents in scaffolding learning, supporting both observational learning and the development of self-efficacy, as proposed by Bandura [8].

Integration of theory and practice

Students perceived simulation as facilitating the integration of theoretical knowledge into clinical practice, particularly in complex contexts [44]. These perceptions reflect one of the central challenges in clinical reasoning education: the transfer of knowledge between theoretical and clinical domains [34]. Rather than demonstrating objective transfer, the findings suggest that simulation provides a space where fragmented knowledge can be mobilised, tested, and reorganised. This process reflects early-stage development described by Benner, in which learners begin to situate abstract knowledge within concrete clinical experiences, supporting the transition from novice to more competent forms of reasoning [2].

Simulation also appeared to create conditions for practising prioritisation and interpretive judgement [45], although these should be understood as perceived rather than measured outcomes. Similarly, reported gains in confidence, autonomy, and self-efficacy reflect subjective learning experiences rather than demonstrable performance improvements [39]. These perceived gains in self-efficacy are consistent with Bandura’s framework, suggesting that mastery experiences, feedback, and social interaction within simulation may strengthen learners’ beliefs in their capacity to make clinical decisions [8].

Learning through error and reflective processes

Learning through error emerged as a key mechanism within simulation. Immediate feedback allowed students to identify gaps, reconsider decisions, and adjust their reasoning, supporting reflective processes and self-regulation [43,45]. These findings suggest that simulation functions as a space for reflective interruption, where students reassess assumptions and refine decision-making in a controlled environment. This iterative process is closely aligned with Tanner’s “reflecting” phase, in which learners evaluate their actions and integrate new insights into future reasoning [4].

Collaborative problem-solving further supported the co-construction of knowledge and the development of both autonomous and interdependent competencies [20]. Self-awareness emerged not as an isolated attribute, but as a process shaped by feedback, peer interaction, and reflection [46]. Such processes also reinforce Bandura’s notion of self-regulation, whereby learners actively monitor and adjust their cognitive strategies in response to feedback and experience [8].

Critical considerations and limitations

Despite the overall positive findings, several critical considerations should be acknowledged. Data were collected within the course context, and participants may have associated the research team with their learning experience, introducing potential social desirability bias. The dual role of researchers as educators may also have influenced both data collection and interpretation, despite efforts to minimise these effects.

The study was conducted within a single institution and cohort, limiting transferability. Furthermore, findings are based on self-reported perceptions and were not triangulated with observational or performance-based data. The absence of quantitative assessment prevents conclusions about actual development of clinical reasoning. In addition, the study does not isolate the effects of simulation from collaborative learning, limiting interpretation of their individual contributions.

Software and pedagogical limitations

While most findings reflected positive perceptions, important limitations were identified, particularly in relation to its suitability for nursing practice. Students noted the absence of autonomous nursing interventions within the simulated scenarios, which may undermine perceptions of the simulator’s alignment with professional reality. Participants also described misalignment between system-generated scores and their reasoning processes, which was perceived as demotivating. This aligns with previous research indicating that automated assessment systems may inadequately capture the complexity of clinical reasoning [47,48]. Such discrepancies may reflect construct underrepresentation, where assessment captures only part of the intended competence [46,47]. From this perspective, the findings highlight the challenge of aligning standardised evaluation systems with the interpretive and context-dependent nature of clinical judgement, as conceptualised by Tanner [4].

These findings suggest that the pedagogical value of simulation depends not only on scenario realism, but also on alignment between assessment and learning objectives [47,48]. The role of the instructor is therefore critical in mediating these limitations and supporting reflective learning [49].

Students also reported pedagogical constraints, including limited exposure to simulation and time for practice. As noted by Theobald et al. [43], such factors can influence learning outcomes, highlighting the importance of intentional pedagogical design.

Implications and future research

From a practical perspective, the findings highlight the importance of integrating virtual simulation within curricula in a way that ensures alignment between learning objectives, assessment, and pedagogical strategies since the early stages of the nursing degree. Automated scoring should be complemented by structured debriefing to support reflection and validation of reasoning processes.

Future research should adopt mixed-methods approaches, combining qualitative insights with objective measures of clinical reasoning. Studies should also seek to disentangle the effects of simulation and collaborative learning, to better understand their individual contributions.

## 5. Conclusions

This study advances understanding of clinical reasoning development in nursing education by offering a process-oriented account of how novice learners begin to structure reasoning within collaborative virtual simulation environments. By focusing on first-year students, the findings illuminate a formative stage that remains underexplored and highlight the importance of early pedagogical experiences in shaping clinical thinking.

The findings indicate that virtual patient simulation functions as a socio-cognitive learning environment that supports the emergence of clinical reasoning through interaction, reflection, and contextualised decision-making. Realism, collaboration, and feedback appear to facilitate engagement with key reasoning processes, including cue recognition, prioritisation, and reflective judgement.

Importantly, the study underscores that the educational value of simulation depends on the alignment between technology, pedagogy, and social interaction. Collaborative learning and guided facilitation play a central role in enabling students to externalise and refine their reasoning.

These findings contribute to a more nuanced understanding of early clinical reasoning development and provide relevant insights for the design of simulation-based learning. In particular, they highlight the need for intentional curricular integration, alignment between learning and assessment, and structured opportunities for reflection. Future research should further explore these processes using mixed-methods approaches.

## Data Availability

The data presented in this study are available on request from the corresponding author. The data are not publicly available due to the length of the article.

## Public Involvement Statement

There was no public involvement in any aspect of this research.

## Guidelines and Standards Statement

This manuscript was drafted against the COREQ—Consolidated Criteria for Reporting Qualitative Research.
